# A ^15^N stable isotope semen label to detect mating in the malaria mosquito *Anopheles arabiensis *Patton

**DOI:** 10.1186/1756-3305-1-19

**Published:** 2008-07-01

**Authors:** Michelle EH Helinski, Rebecca C Hood, Doris Gludovacz, Leo Mayr, Bart GJ Knols

**Affiliations:** 1 Agency's Laboratories Seibersdorf, International Atomic Energy Agency (IAEA), A-2444 Seibersdorf, Austria; 2 Laboratory of Entomology, Wageningen University and Research Centre, P.O. Box 8031, 6700 EH Wageningen, The Netherlands; 3 Department of Entomology, Ithaca campus, Cornell University. 3136 Comstock Hall, Ithaca, NY 14853-2601, USA

## Abstract

In previous studies it was determined that the stable isotope 13-carbon can be used as a semen label to detect mating events in the malaria mosquito *Anopheles arabiensis*. In this paper we describe the use of an additional stable isotope, 15-nitrogen (^15^N), for that same purpose. Both stable isotopes can be analysed simultaneously in a mass spectrometer, offering the possibility to detect both labels in one sample in order to study complex and difficult-to-detect mating events, such as multiple mating. ^15^N-glycine was added to larval rearing water and the target enrichment was 5 atom% ^15^N. Males from these trays were mated with unlabelled virgin females, and spiked spermathecae were analysed for isotopic composition after mating using mass spectrometry. Results showed that spermathecae positive for semen could be distinguished from uninseminated or control samples using the raw δ^15^N‰ values. The label persisted in spermathecae for up to 5 days after insemination, and males aged 10 days transferred similar amounts of label as males aged 4 days. There were no negative effects of the label on larval survival and male longevity. Enrichment of teneral mosquitoes after emergence was 4.85 ± 0.10 atom% ^15^N. A threshold value defined as 3 standard deviations above the mean of virgin (*i.e. *uninseminated spermathecae) samples was successful in classifying a large proportion of samples correctly (*i.e. *on average 95%). We conclude that alongside ^13^C, ^15^N can be used to detect mating in *Anopheles *and the suitability of both labels is briefly discussed.

## Findings

In this paper, we investigated the use of the stable isotope 15-nitrogen (^15^N) as a semen label for mosquito mating. Recent studies undertaken in our laboratory have documented the successful application of stable isotopes as a population marker in the context of genetic control studies [1], and the suitability of carbon-13 (^13^C) as a semen label was established [2]. The use of an additional isotope would allow dual-labelling of two groups of males to determine for instance paternity in competition experiments, or to study multiple mating events. Because the isotopes ^13^C and ^15^N can be analysed simultaneously in one sample, the use of ^15^N for the labelling of semen was investigated.

For all experiments the Dongola strain of *Anopheles arabiensis *Patton was used. Rearing techniques were identical to those described by Helinski *et al. *[2]; only a slightly larger volume (*i.e. *1.5 L) of water was used in the larval trays. Ninety-eight atom% ^15^N-glycine (NLM-202-1, Cambridge Isotope Laboratories Inc, Andover, MA, USA) was used as a label. Mosquitoes were exposed to the label in the larval stage on the day the L1 larvae were introduced. The level of enrichment was 5 atom% ^15^N (*i.e. *5% of all the nitrogen in the diet was ^15^N) and the amount of ^15^N-glycine added was based on the total amount of nitrogen present in the diet. Until pupation, 1250 mg (0.25 mg × 500 larvae × 10 days) of larval food was added to the tray; consisting of approx. 7.3% nitrogen, thus 90.75 mg of nitrogen was added. To achieve the enrichment of 5 atom% ^15^N, 4.63 mg of ^15^N was required ((90.75 × 0.05))/0.98). Glycine consists for approx. 20% of nitrogen, thus 23.48 mg of 98 atom% ^15^N-glycine was added to the larval trays. A solution was made containing 117.40 mg in 250 ml H_2_O and each tray received 50 ml. Solutions were kept at 4°C.

**Figure 1:**
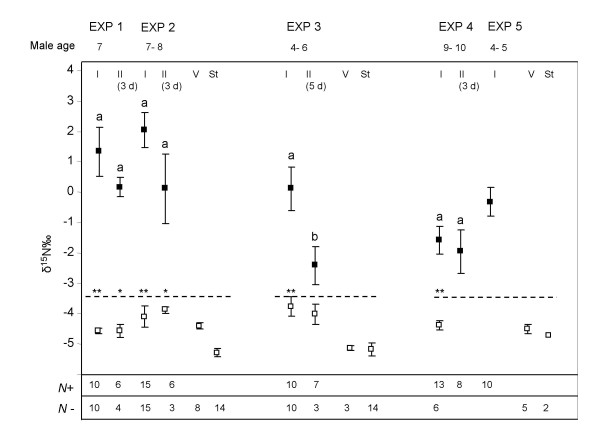
**Mean ± s.e.m. δ^15^N‰ values of spermathecae of inseminated (filled symbols) and uninseminated (open symbols) females from experiments 1–5.** Male age at the start of the experiment is given. *N *is the number of spermathecae analysed for inseminated (+) and uninseminated (-) females. The dotted line indicates the threshold value of 3 s.d. above mean δ^15^N‰ of virgin females. Virgin (V) and Standard (St) samples are included. Dissection treatments are: I: females dissected immediately after mating; II: females isolated and dissected 3–5 days after mating (with the number of days used added in between brackets). Values with different letters are significantly different at p < 0.05 with comparisons made between dissection treatments I and II for each experiment. Asterisks indicate significant difference between spermathecae of inseminated and uninseminated females for each treatment at * p < 0.05, and ** p < 0.01 (comparisons made with independent t-tests or Mann-Whitney U tests).

A total of five separate mating experiments were performed each with different batches of ^15^N-labelled males (Fig. 1). All mosquitoes used in the experiments were maintained as virgins before mating by the sexing of adults within 18 hrs after eclosion. After mating, females were either dissected immediately (*i.e. *the following day) at the end of the mating period (I); or isolated for dissection at a later stage to assess the persistence of the label in the spermathecae (II). Males varied in age between 4 to 10 days at the start of the experiments (see Fig. 1), and mating lasted for 1–3 nights. Survival of larvae from labelled or control (*i.e. *unlabelled) treatments was determined for three experiments, and in each experiment two trays per treatment were used. To determine adult male longevity, newly emerged males of the ^15^N-labelled tray and the control tray were placed in a standard rearing cage to monitor survival (*N *= 25) and this was replicated twice using males from different experiments (in one of the experiments 3 duplicates were performed). Mortality was scored regularly until all males had died.

Sample preparation was similar to that described in Helinski *et al. *[2]. The amount of nitrogen present in the spermatheca was below the detection limit of the mass spectrometer setup (approx. > 20 μg). Samples were therefore "spiked" with 10 μl of a standard ammonium sulphate solution containing ~20 μg of nitrogen [3]. Virgin (*i.e. *spermathecae from virgin females) and standard samples (*i.e. *tin cup containing just the spike on quartz paper) were included. Whole body analyses were performed on teneral mosquitoes to determine their overall level of enrichment. Sample analysis [4] and interpretation were similar to Helinski *et al. *[2]. The δ^15^N‰ values reported are referenced to the international reference standard for nitrogen, *i.e. *atmospheric nitrogen or AIR. Samples were analysed at the International Atomic Energy Agency.

Prior to analyses, data were checked for normality and the appropriate tests were performed (*i.e. *General Linear Models (GLMs) with planned contrasts (Tukey's HSD) or independent *t*-tests for normally distributed data, and Mann-Whitney U tests or Kruskal-Wallis tests with Bonferroni correction for not-normally distributed data). A threshold value to distinguish labelled spermathecae from unlabelled spermathecae was defined as 3 standard deviations (s.d.) above the mean δ^15^N (‰) value of the reference standard [5], in our case virgin females. Longevity of males was analysed using Kaplan-Meier survival analyses and Mantel-Cox log-rank tests with Bonferroni correction. All tests were used two-sidedly and were performed using the SPSS software version 12 (SPSS Inc., Chicago, USA).

Some technical difficulties with the mass spectrometer caused variance in standard reference samples between experimental runs in experiment three and to a lesser extent in experiments 4 and 5. These inconsistencies probably have resulted in a few erroneous δ^15^N‰ values for some samples. As we deemed it inappropriate to simply remove unwanted results, all data points were included for completeness and statistical analysis. However, significantly lower values were reported for spermathecae from virgin females from experiment three compared to the values in the other experiments (F_(2,13) _= 6.42, p < 0.05); thus the threshold value in experiment three was replaced by the value from experiments 1–2. Means throughout the text are reported ± s.e.m.

Labelling with ^15^N-glycine in the larval stage resulted in the detection of ^15^N enrichment in spermathecae when labelled males were mated with unlabelled females (Fig. 1). When females were dissected immediately after mating (I), mean δ^15^N‰ values of spermathecae for inseminated females were significantly higher than values for uninseminated females for all experiments (Mann-Whitney U or independent t-tests, Fig. 1). When females were isolated after mating and dissected at later intervals (II), δ^15^N‰ values of spermathecae of inseminated females were still significantly higher compared to values for spermathecae of uninseminated females in all experiments ((Mann-Whitney U or independent t-tests, Fig. 1), with the exception of experiment three (*t*(8) = 1.59, p > 0.05). However, this was the result of one low δ^15^N‰ value for a spermatheca of an inseminated female in the dataset; without it spermathecae of inseminated females were significantly higher compared to uninseminated ones (*t*(7) = 2.87, p < 0.05). Isolation of the females did not result in significantly lower mean δ^15^N‰ values compared to the values observed after immediate dissection for each experiment (Mann-Whitney U tests; data not shown), except for experiment three (*t*(15) = 2.54, p < 0.05; Fig. 1); again after removal of this one low value no significant differences were observed (*t*(7) = 2.06, p > 0.05).

Males used in the experiments varied in age between 4 to 10 days, however, the amount of label transferred to females was similar for young (exp. 5) and old (exp. 4) males (*U *= 36, r = -0.38, p > 0.05). The δ^15^N‰ values of uninseminated spermathecae were similar to samples from virgin females; and both were significantly higher than the standard samples (*i.e. *without spermathecae; *X*^2 ^= 35.11; df = 2, p < 0.01). The threshold values could be used to identify inseminated spermathecae from uninseminated spermathecae in the large majority of cases. Some false positives and negatives were observed in the dataset, but overall 95 ± 2% of the samples were classified correctly.

Only a limited number of teneral mosquitoes (*N *= 4; 2 for each sex) were analysed to determine the enrichment level, which was on average 4.85 ± 0.10 atom% ^15^N.

The addition of ^15^N-labelled glycine had no effect on larval survival or development, and survival of labelled larvae (84 ± 8%) was similar to survival of larvae in the control trays (87 ± 5%; *t*(4) = 0.32, p > 0.05). Adult male longevity was not affected by the ^15^N-glycine label, and a higher or similar longevity for ^15^N males was observed compared to unlabelled males (data not shown). After 1 night of mating approximately 50% of females were inseminated by labelled males.

The data presented here confirm that ^15^N-glycine can be used as a semen label to detect mating; sufficient amounts of label were transferred to distinguish inseminated females from uninseminated ones. Isolation of the females after mating did not result in a loss of label. The label persisted in the males for up to ten days of age; and similar levels of label were transferred compared to younger males. Even though some problems were observed with the sample analysis, the large majority of samples were classified correctly using the threshold value. The label appeared to have no influence on larval development or survival and adult male longevity. In addition, mating ability of labelled males was good and similar results were observed with ^13^C-labelled males [4]. In terms of costs, ^15^N labelling is preferable over ^13^C as smaller amounts of label are required to achieve a similar level of enrichment. The addition of ^15^N-glycine resulted in less problems with larval development compared to results sometimes observed for ^13^C-glucose [6]. However, the sample analyses tends to be easier for ^13^C compared to ^15^N when working with the low quantities used in these studies, and this probably accounted for some of the difficulties observed with the sample analyses. It is therefore recommended that a slightly higher level (e.g. double the amount) of ^15^N label is used in further experiments.

In conclusion, the current work has shown that labelling of semen with nitrogen was successful and thus ^15^N and ^13^C can be used simultaneously as a dual-labelling system [6].

## Competing interests

The authors declare that they have no competing interests.

## Authors' contributions

MEHH designed and performed the experiments, analysed the data and wrote the manuscript; RCH co-designed the experiment, assisted in data analysis, and edited the manuscript; DG and LM analysed the samples; BGJK supervised the work and the edited the manuscript. All authors read and approved the final version of the manuscript.
